# A Two-Step Regional Ionospheric Modeling Approach for PPP-RTK

**DOI:** 10.3390/s24072307

**Published:** 2024-04-05

**Authors:** Zhenyu Xu, Changsheng Cai, Lin Pan, Wujiao Dai, Bei He

**Affiliations:** 1 School of Geosciences and Info-Physics, Central South University, Changsha 410083, Chinalinpan@csu.edu.cn (L.P.); wjdai@csu.edu.cn (W.D.);; 2 State Key Laboratory of Geo-Information Engineering, Xi’an 710054, China

**Keywords:** GNSS, ionospheric delay, PPP-RTK, polynomial fitting model, Kriging interpolation

## Abstract

In the precise point positioning/real-time kinematic (PPP-RTK) technique, high-precision ionospheric delay correction information is an important prerequisite for rapid PPP convergence. The commonly used ionospheric modeling approaches in the PPP-RTKs only take the trend term of the ionospheric total electron content (TEC) variations into account. As a result, the residual ionospheric delay still affects the positioning solutions. In this study, we propose a two-step regional ionospheric modeling approach that involves combining a polynomial fitting model (PFM) and a Kriging interpolation (KI) model. In the first step, a polynomial fitting method is used to model the trend term of the ionospheric TEC variations. In the second step, a KI method is used to compensate for the residual term of the ionospheric TEC variations. Datasets collected from continuously operating reference stations (CORSs) in Hunan Province, China, are used to validate the PFM/KI method by comparing with a single PFM method and a combined PFM and inverse distance weighting interpolation (IDWI) method. The experimental results show that the two-step PFM/KI modeled ionospheric delay achieves an average root mean square (RMS) error of 1.8 cm, which is improved by about 48% and 23% when compared with the PFM and PFM/IDWI methods, respectively. Regarding the positioning performance, the PPP-RTK with the PFM/KI method takes an average of 1.8 min or 4.0 min to converge to a positioning accuracy of 1.3 cm or 2.5 cm in the horizontal and vertical directions, respectively. The convergence times are decreased by about 18% and 14% in the horizontal direction and 9% and 5% in the vertical direction over the PFM and the PFM/IDWI methods, respectively.

## 1. Introduction

The Global Navigation Satellite System (GNSS) precise point positioning (PPP) technique has been widely used due to its high accuracy, easy operation, and independence of base stations [1,2]. Although the PPP technique can achieve decimeter-level or centimeter-level positioning accuracy globally with only a single receiver, it needs a long convergence time of about half an hour before achieving the desirable accuracy, severely restricting its use in real-time application fields [3,4]. To improve the PPP convergence performance, PPP ambiguity resolution (AR) techniques are developed by fixing the float ambiguities [5]. Although the PPP-AR techniques can speed up the PPP convergence, they still need a long initialization time before ambiguities can be fixed. An effective processing strategy for reducing the PPP convergence time is to use the additional atmospheric delay information. Wübbena et al. [6] proposed the precise point positioning/real-time kinematic (PPP-RTK) technique, which uses known reference station coordinates to calculate the atmospheric delay in real-time and then broadcast the atmospheric correction information to users, to realize real-time PPP in the service area. With the aid of the outer atmospheric delay information, the PPP-AR can be obtained instantly [7].

In PPP-RTKs, the quality of ionospheric products has a direct impact on the users’ positioning performance [8]. As a publicly available product from the International GNSS Service (IGS), the global ionospheric map (GIM) can be used to constrain ionospheric parameters, to shorten the initialization time of PPP. However, due to the limited accuracy of the GIM, the PPP convergence time is still about 15 min [9]. Furthermore, the GIM product has an accuracy of only 2–8 total electron content units (TECU), which cannot meet the accuracy required for PPP-RTKs of 0.7 TECU (equivalent to a ranging error of 11.4 cm for a GPS L1 frequency signal) [10]. Thus, regional ionospheric models have to be built to satisfy the demand for accuracy. Conventionally, the measured slant ionospheric total electron content (TEC) values for all observed satellites at the reference stations during a period are projected in the vertical direction to establish the regional ionospheric model. When the users use the ionospheric products, the vertical total electron content (VTEC) is projected back to the slant total electron content (STEC) to correct the ionospheric delay errors. However, in the process of projection, it is inevitable that accuracy loss will occur [11]. To meet high-accuracy and real-time demands, the STEC is straightforwardly utilized without projecting to establish the regional ionospheric model on a satellite-by-satellite basis in PPP-RTKs.

So far, two kinds of regional ionospheric modeling approaches to PPP-RTKs have been developed. One is the spatial interpolation approach, which uses an interpolation function to calculate the ionospheric delay based on the spatial relationship between the user stations with unknown STECs and the reference stations with known STECs. As long as there are enough reference stations around the user stations, the interpolation calculation can be carried out with an interpolation accuracy depending on the known STEC accuracy and their spatial correlation. This spatial interpolation approach can usually acquire higher-accuracy ionospheric delay information at user stations but simultaneously has higher requirements for the network bandwidth in PPP-RTK practical applications. The commonly used interpolation methods include the inverse distance weighting interpolation (IDWI) method [12], the Kriging interpolation (KI) method [13], and the linear combination method [14]. These methods can be applied in a region with varying accuracies depending on the coverage and density of the reference station network. The other is the fitting function modeling approach, which utilizes a function to fit the trend term of the spatial ionospheric TEC variations based on an optimal estimation theory. After the model coefficients are obtained, the ionospheric delay correction at the user stations can be derived. Although the fitting function modeling approaches have high requirements for the uniformity of reference station distribution, they are conducive to extrapolation. The commonly used fitting function models include the spherical harmonic function model [15], the trigonometric series model [16], and the polynomial fitting model (PFM). Among them, the PFM is more widely used due to its easy implementation [17]. It models the STEC on a single layer as a function of the longitude and latitude differences of the reference stations and the center point of the modeled area. The polynomial orders depend on the scale of the reference network [18]. Since only the polynomial coefficients are needed to transmit to user stations in order to calculate the ionospheric delay correction, the communication burden on the server side is small. Despite this, its disadvantage is also obvious. The residual ionospheric delay is probably large in a local area due to the uneven geographical distribution of reference stations or inconsistent STEC accuracy at reference stations.

The above two kinds of regional ionospheric modeling approaches have their advantages and disadvantages. To combine their benefits, Cui et al. [19] developed a hierarchical ionospheric augmentation method by combining the PFM and the IDWI. Compared with a single IDWI method, the transmission of the ionospheric delay corrections can be reduced by 61%. Compared with a single PFM method, the convergence time of the position coordinates can be improved by 68%. Although the augmentation method has obvious superiority, it only uses the distance as the weight for interpolation calculation in the IDWI, which is improper when the reference stations cannot be evenly distributed around the user stations. Differing from the IDWI method, the KI method explicitly takes the spatial correlation structure into account, which allows for providing more accurate estimates in areas where stations are unevenly distributed or where there is a complex spatial pattern. Thus, weights can be assigned according to the spatial relationship between sampling points, to obtain more accurate interpolation results [20]. To make the most of its advantages, we propose a two-step regional ionospheric modeling approach based on the PFM and KI. The first step is to establish a PFM. In the second step, the KI method is used to compensate for the STEC unmodeled errors at the user position. PPP-RTK experiments are carried out to fully evaluate the proposed two-step regional ionospheric modeling approach.

This paper is structured as follows. A two-step regional ionospheric modeling approach is presented in Section 2. The PPP processing strategies are presented in Section 3. The regional ionospheric modeling accuracy and the PPP-RTK positioning performance are then evaluated in Section 4. Finally, some conclusions are drawn in Section 5.

## 2. Regional Ionospheric Modeling Approach

In PPP-RTKs, the regional ionospheric model is established in two processes. In the first process, the high-precision slant ionospheric delay for each visible satellite is extracted from the regional network using an undifferenced and uncombined (UDUC) PPP-AR method [21]. In the second process, the extracted slant ionospheric delays at reference stations are used to establish an epoch-wise ionospheric model for each satellite, to acquire the ionospheric delay information at user stations.

### 2.1. Slant Ionospheric Delay Extraction

Reference stations in a regional network are used to acquire the ionospheric delay information. The slant ionospheric delay for a satellite s at a reference station r can be estimated using a UDUC PPP model as shown below.

(1) $$\left\{\begin{array}{c} P_{r,i}^{s}=\rho_{r}^{s}+cdt_{r}-cdt^{s}+\gamma_{i}I_{r,1}^{s}+T_{r}^{s}+d_{r,i}+d_{i}^{s}+\varepsilon_{P_{r,i}}^{s}\\ L_{r,i}^{s}=\rho_{r}^{s}+cdt_{r}-cdt^{s}-\gamma_{i}I_{r,1}^{s}+T_{r}^{s}+N_{r,i}^{s}+b_{r,i}+b_{i}^{s}+\varepsilon_{L_{r,i}}^{s} \end{array}\right.$$

where $P_{r,i}^{s}$ is the code observation in meters, $L_{r,i}^{s}$ represents the carrier phase observation in meters, the subscript i indicates the frequency of the corresponding observation, $\rho_{r}^{s}$ is the geometric range from the satellite s to the receiver r in meters, c is the speed of light in meters per second; $dt_{r}$ and $dt^{s}$ represent the receiver and satellite clock offsets in seconds, respectively; $\gamma_{i}=\frac{f_{1}^{2}}{f_{i}^{2}}$ is ionospheric conversion coefficient, and f is the signal frequency; $I_{r,1}^{s}$ denotes the slant ionospheric delay at the first frequency in meters; $T_{r}^{s}$ denotes the tropospheric delay in meters; $N_{r,i}^{s}$ is the carrier phase ambiguity in meters; $d_{r,i}$ and $d_{i}^{s}$ represent the code hardware delay of the receiver and satellite in meters, respectively; $b_{r,i}$ and $b_{i}^{s}$ represent the carrier phase hardware delay of the receiver and satellite in meters, respectively; $\varepsilon_{P_{r,i}}^{s}$ and $\varepsilon_{L_{r,i}}^{s}$ are the code and carrier phase observation noises in meters, respectively. Other errors such as the satellite and receiver antenna phase center offsets (PCOs) and phase center variations (PCVs), relativistic effect, tidal loadings, and antenna phase windup also need to be considered in PPP, although they are not modeled in Equation (1). Their handling strategy can be referred to Kouba and Héroux [2].

In the UDUC PPP, precise ephemeris products are used to mitigate the satellite orbit and clock errors. Since the satellite clock products are generated based on a specific observation combination such as a GPS L1/L2 ionosphere-free combination, the clock offset corrections inevitably include a frequency-dependent code hardware delay. Thus, the code observations on a specific frequency need to be treated with satellite differential code bias (DCB) corrections to keep up consistency with precise clock products [22]. After applying all kinds of error corrections, the UDUC PPP observation equation can be expressed as [23].

(2) $$\left\{\begin{array}{c} p_{r,i}^{s}=\mu_{r}^{s}\cdot X_{r}+c\bar{dt_{r}}+\gamma_{i}\cdot {\bar{I}}_{r,1}^{s}+m_{r}^{s}\cdot T_{r,ZWD}+\theta_{i}\cdot \psi_{i}\\ l_{r,i}^{s}=\mu_{r}^{s}\cdot X_{r}+c\bar{dt_{r}}-\gamma_{i}\cdot {\bar{I}}_{r,1}^{s}+m_{r}^{s}\cdot T_{r,ZWD}+{\bar{N}}_{r,i}^{s} \end{array}\right.,\;\theta =\left\{\begin{array}{c} 1,\;i\geq 3\\ 0,\;i<3 \end{array}\right.$$

(3) $$\left\{\begin{array}{c} c\bar{dt_{r}}=cdt_{r}+a_{12,1}\cdot d_{r,1}+a_{12,2}\cdot d_{r,2}\\ {\bar{I}}_{r,1}^{s}=I_{r,1}^{s}-a_{12,2}\cdot \left(d_{r,2}-d_{r,1}\right)\\ \psi_{i}=\left(-\gamma_{i}\cdot a_{12,2}-a_{12,1}\right)\cdot d_{r,1}-a_{12,2}\cdot \left(1-\gamma_{i}\right)\cdot d_{r,2}+d_{r,i}\\ {\bar{N}}_{r,i}^{s}=N_{r,i}^{s}+b_{r,i}+b_{i}^{s}-d_{i}^{s}-\left(a_{12,1}-\gamma_{i}\cdot a_{12,2}\right)\cdot d_{r,1}-a_{12,2}\cdot \left(1+\gamma_{i}\right)\cdot d_{r,2} \end{array}\right.$$

where $p_{r,i}^{s}$ and $l_{r,i}^{s}$ represent the observed-minus-computed pseudorange and carrier phase observations in meters, respectively; $\mu_{r}^{s}$ is the unit vector in the line-of-sight direction; $X_{r}$ is the three-dimensional coordinate of the receiver in meters; $c\bar{dt_{r}}$ is the receiver clock offsets parameter that absorbs the receiver code hardware delay in meters ($a_{12,1}=f_{1}^{2}/\left(f_{1}^{2}-f_{2}^{2}\right),a_{12,2}=-f_{2}^{2}/\left(f_{1}^{2}-f_{2}^{2}\right)$); ${\bar{I}}_{r,1}^{s}$ is the slant ionospheric delay that absorbs the receiver code hardware delay in meters; $T_{r,ZWD}$ is the tropospheric zenith wet delay (ZWD) in meters; $\psi_{i}$ is the inter-frequency bias parameter in meters, which exists only in the pseudorange observation equation of the multi-frequency PPP; and ${\bar{N}}_{r,i}^{s}$ is the ambiguity that absorbs the code and phase hardware delay at the satellite and receiver in meters. The unknown parameters in the UDUC PPP functional model include three station coordinates, one receiver clock offset, one tropospheric zenith wet delay (ZWD), slant ionospheric delay parameters equal to the number of observed satellites, and ambiguity parameters. The total tropospheric delay is divided into hydrostatic and wet components. The zenith hydrostatic delay is corrected by a tropospheric delay model, and only the ZWD is estimated as an unknown parameter.

A reliable ambiguity-fixed solution is the key to guaranteeing the accuracy of the estimated ionospheric delay. To restore the integer characteristics of ambiguities, the influence of the receiver hardware delay is eliminated by an inter-satellite single-difference operation. The uncalibrated phase delay (UPD) product derived from a fractional cycle bias estimation method is used to fix float ambiguities to integer ambiguities [23]. Because of the strong correlation of the phase ambiguities from different satellites, the least-squares ambiguity decorrelation adjustment (LAMBDA) method [24,25] is used to search for and fix ambiguities. Once the ambiguities are successfully fixed, an integer ambiguity constraint condition is added to the observation equation as pseudo-observables to restrain the fractional part of the ambiguity [26]. After the UDUC PPP-AR converges, the slant ionospheric delay estimates are extracted.

### 2.2. Two-Step Regional Ionospheric Modeling

Given the complexity of the ionospheric spatial variation, we propose a two-step regional ionospheric modeling approach in which a polynomial function is first utilized to model the trend term of the ionospheric variation, and then a KI method is employed to compensate for the residual ionospheric delay. The ionospheric delay is assumed to concentrate on a thin layer at an altitude of about 350~450 km from the ground [27]. When the satellite signal transmits to stations via the thin layer, the relative positions of the ionospheric pierce points (IPPs) are similar to the relative positions of reference stations for a certain satellite. Thus, the reference station positions can be used to establish the regional ionospheric model instead of the IPP positions for simplicity. In this study, the ionospheric delay for a single satellite is expressed as a polynomial function concerning the longitude and latitude differences between the reference stations and the center point of the modeling region, as shown below.

(4) $$I_{r}^{s}=a_{0}+a_{1}\cdot \Delta \varphi_{r}+a_{2}\cdot \Delta \lambda_{r}+a_{3}\cdot \Delta \varphi_{r}^{2}+a_{4}\cdot \Delta \lambda_{r}^{2}+a_{5}\cdot \Delta \varphi_{r}\cdot \Delta \lambda_{r}$$

where $I_{r}^{s}$ is the slant ionospheric delay of the satellite s at the station r; $\Delta \varphi_{r},\;\Delta \lambda_{r}$ are the latitude and longitude differences of the station and center point of the modeling region, respectively; and $a_{i}\left(i=0,1,\ldots ,5\right)$ are the coefficients of the polynomial function.

Since the extracted slant ionospheric delay contains the receiver hardware delay, an inter-satellite single-difference operation is performed to remove the receiver hardware delay, as shown below.

(5) $$\begin{array}{c} ∆{\bar{I}}^{s,s_{0}}={\bar{I}}^{s}-{\bar{I}}^{s_{0}}=a_{0}^{s,s_{0}}+a_{1}^{s,s_{0}}\cdot \Delta \varphi_{r}+a_{2}^{s,s_{0}}\cdot \Delta \lambda_{r}\\ +a_{3}^{s,s_{0}}\cdot \Delta \varphi_{r}^{2}+a_{4}^{s,s_{0}}\cdot \Delta \lambda_{r}^{2}+a_{5}^{s,s_{0}}\cdot \Delta \varphi_{r}\cdot \Delta \lambda_{r} \end{array}$$

where the superscript $s_{0}$ denotes a reference satellite.

After the polynomial function is used to model the trend term of the ionospheric delay, the residual term still exists due to the irregular distribution of the ionospheric delay in space. To compensate for the residual term, a KI method [28] is further used to reduce the effect of the residual ionospheric delay when the residual value is significant. The KI model is shown below.

(6) $$\hat{Z}\left(r_{0}\right)=\sum_{i=1}^{N}\omega_{i}Z\left(r_{i}\right)$$

where $\hat{Z}\left(r_{0}\right)$ is the residual ionospheric delay at the position $r_{0}$; and $\omega_{i}\left(i=1,2,\ldots ,N\right)$ is the weight of the corresponding sampling point, which depends on the semi-variogram used to describe spatial dependence and variability. The semi-variogram is defined as $\gamma \left(h\right)=\frac{1}{2}E{\left[Z\left(r\right)-Z\left(r+h\right)\right]}^{2}$, which reveals the randomness and structural characteristics of regionalized variables. In this study, an exponential model is employed to calculate the semi-variogram, as shown in Equation (7).

(7) $$\gamma \left(h\right)=\left\{\begin{array}{cc} C_{0} & ,\;h=0\\ C_{0}+C\left(1-e^{-\frac{h}{a}}\right) & ,\;0<h\leq 3a\\ C_{0}+C & ,\;h>3a \end{array}\right.$$

where $C_{0}$ is a nugget, C is a partial sill, $C_{0}+C$ is a sill; h is the distance between stations, and 3a is the range, which reflects the distance at which the variogram tends to be stable. Through the fitted semi-variogram, the weight of sampling points can be calculated by Equation (8).

(8) $$\left[\begin{array}{c} \omega_{1}\\ \omega_{2}\\ \vdots \\ \omega_{N}\\ m \end{array}\right]={\left[\begin{array}{ccccc} \gamma_{11} & \gamma_{12} & \cdots & \gamma_{1N} & 1\\ \gamma_{21} & \gamma_{22} & \cdots & \gamma_{2N} & 1\\ \vdots & \vdots & \ddots & \vdots & 1\\ \gamma_{N1} & \gamma_{N2} & \cdots & \gamma_{NN} & 1\\ 1 & 1 & \cdots & 1 & 0 \end{array}\right]}^{-1}\left[\begin{array}{c} \gamma_{10}\\ \gamma_{20}\\ \vdots \\ \gamma_{N0}\\ 1 \end{array}\right]$$

where $\gamma_{ij}$ denotes the semi-variogram between positions $r_{i}$ and $r_{j}$; m is the Lagrange multiplier; and N is the number of the sampling points. After solving the weight $\omega_{i}\left(i=1,2,\ldots ,N\right)$ of the sampling points, the residual ionospheric delay at the position $r_{0}$ can be calculated, and then the user’s final ionospheric delay correction can be obtained by combining the trend term derived from Equation (5) and the residual term derived from Equation (6).

**Figure 1:**
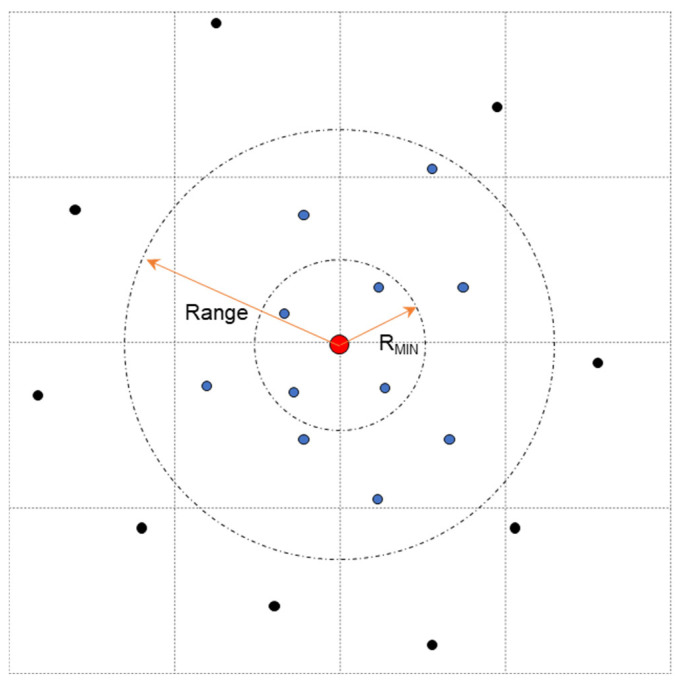
Search diagram of sampling points in Kriging interpolation (KI). The red dot represents the point with the ionospheric delay residual to be estimated. The blue dots represent the sampling points. The black dots represent the excluded points.

To estimate the ionospheric delay residuals at position $r_{0}$, a set of sampling points must reasonably be selected within a certain area. This is illustrated in Figure 1, where the big circle indicates the KI area. The red center point is the position $r_{0}$, which is used to acquire ionospheric delay residuals. The sampling points within the big circle are represented by the blue color, and the sampling points outside the big circle are represented by the black color. The ‘Range’ and ‘R_MIN_’ are the range of the variogram and the minimum search radius, respectively. They are usually set according to the variation degree of the ionospheric TEC in space of the area. Generally, R_MIN_ is smaller than the half of the range. This study used a step-by-step search method to search for the sampling points. First, the search radius was set to R_MIN_. If the number of qualified sampling points was less than the minimum number of targets, the search radius was gradually enlarged to the ‘Range’ until the number of qualified sampling points satisfied the demand for targets. At the end of the search, if the number of sampling points could not satisfy the demand for targets, then interpolation compensation could not be performed at the user position. It is worth noting that not all positions needed interpolation compensation. If the residual terms were smaller than a certain threshold in a local area, it was not necessary to apply the KI. In this case, only the polynomial fitting method was used to acquire the ionospheric delay information and no further interpolation compensation was needed. From this perspective, the two-step regional ionospheric modeling method cannot only reduce the communication burden but also ensure the accuracy of the ionospheric delay information at the user position.

## 3. PPP Processing Strategy

On the server side, UDUC PPP-AR is performed at each reference station to extract the high-precision slant ionospheric delay, and then the two-step regional ionospheric delay model is established for each visible satellite. On the user side, the inter-satellite single-difference slant ionospheric delay at the user’s position is acquired from the server. Based on the UDUC PPP model described in Equation (1), a pseudo-observable equation of the single-difference ionospheric delay is added to the UDUC PPP observation equations to constrain the ionospheric delay parameters, so that the ionospheric parameters and the ambiguity parameters can be quickly separated from each other to achieve a rapid positioning solution convergence. The UDUC PPP processing strategy at the server and user sides is provided in Table 1.

## 4. Results and Analysis

The PFM/KI ionospheric model accuracy was first evaluated with a comparison against the PFM and PFM/IDWI models, and then the PPP positioning performance with ionospheric constraints on the user side was investigated.

### 4.1. Data Description

To validate the proposed two-step PFM/KI regional ionospheric model, datasets at 103 reference stations from continuously operating reference stations (CORSs) in Hunan Province of China on 31 December 2018 were used to evaluate the PFM/KI model with a comparison against the PFM-only and PFM/IDWI models. The searching procedure for sampling points in the IDWI method is similar to that in the KI method, and 12 stations were selected as user stations to test the PPP-RTK positioning performance on a simulated kinematic processing mode. Most of the reference stations are equipped with Trimble NetR9 and LEICA GR10 GNSS receivers and antennas of TRM57971.00 and LEIAR25.R4. Their observations are output in the Receiver INdependent EXchange (RINEX) 2.11 version file format, and only GPS observations are available.

**Figure 2:**
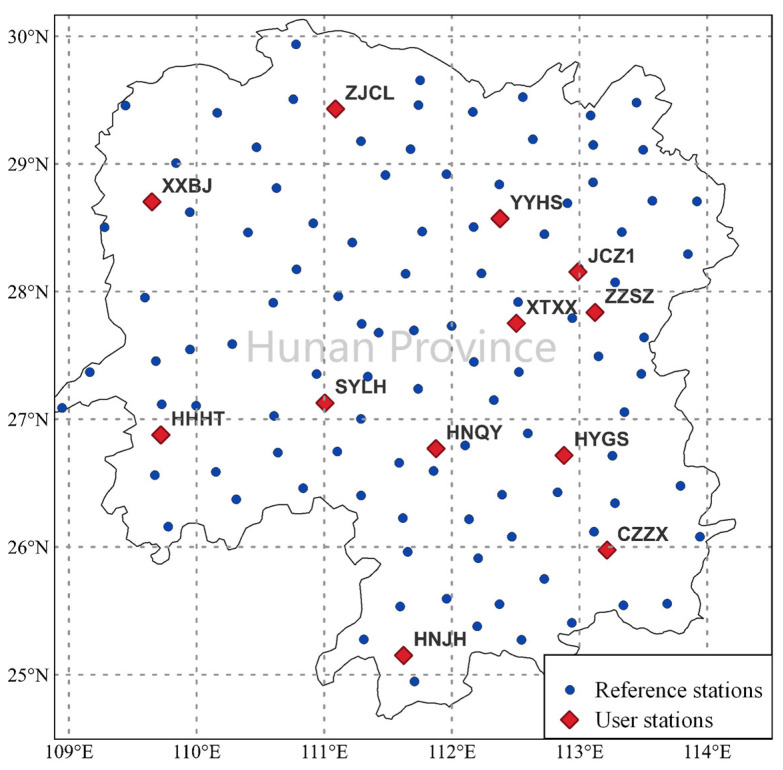
Geographical distribution of reference stations and user stations.

The distances between reference stations are 20~70 km, and the distances between the user stations and the reference stations are about 30~60 km. The geographical distributions of the reference stations and the user stations are shown in Figure 2. The blue dots represent the reference stations while the red diamond-shaped dots denote the user stations. All observations have a sampling interval of 30 s. The epoch-wise PFM/KI model is established on a single-satellite basis.

### 4.2. Ionospheric Model Accuracy

**Figure 3:**
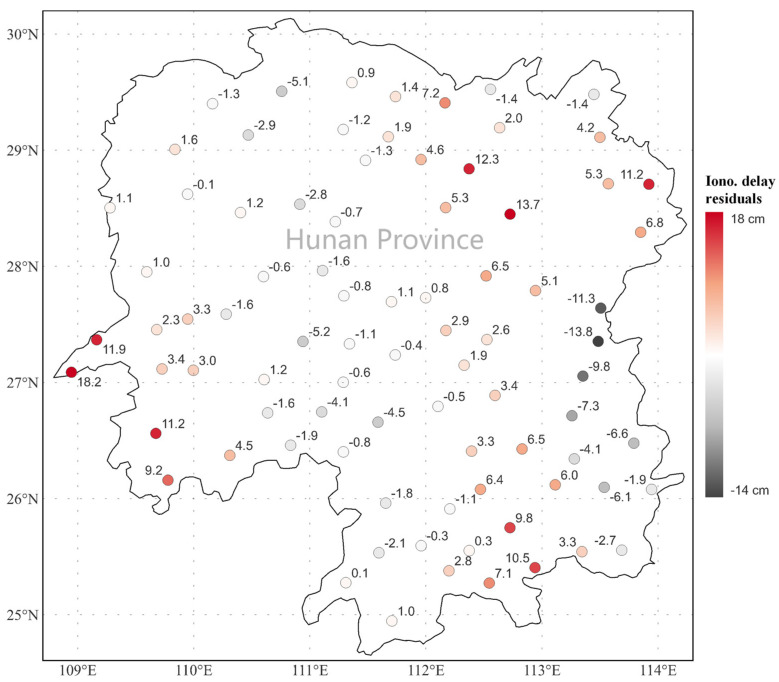
Geographical distribution of residual ionospheric delay after applying the polynomial fitting model (PFM) at GPS time 16:00 for satellite G07.

After applying the PFM, the residual ionospheric delay at each reference station can be obtained according to the difference between the extracted ionospheric observations and the model fitting values. As an example, Figure 3 shows the residual ionospheric delay values at each reference station for GPS satellite PRN 07 at GPS time 16:00. The spatial correlation between stations is obvious. The residual ionospheric delays are typically at 0~10 cm. Due to the boundary effect, the residual values near the boundary are generally larger. However, there are some exceptions. The residual values of two stations in the northeast corner are 12.3 cm and 13.7 cm, which are larger than the residual values of stations near the boundary. This might be caused by the irregular spatial variability of the ionospheric TEC. Therefore, it is necessary to further apply the KI method to compensate for the residual term.

**Figure 4:**
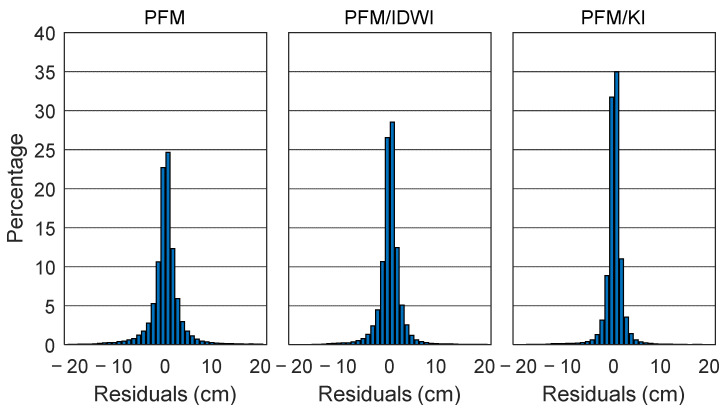
Residuals distribution of PFM, PFM/inverse distance weighting interpolation (IDWI), and PFM/KI ionospheric models for all satellites at all reference stations.

**Figure 5:**
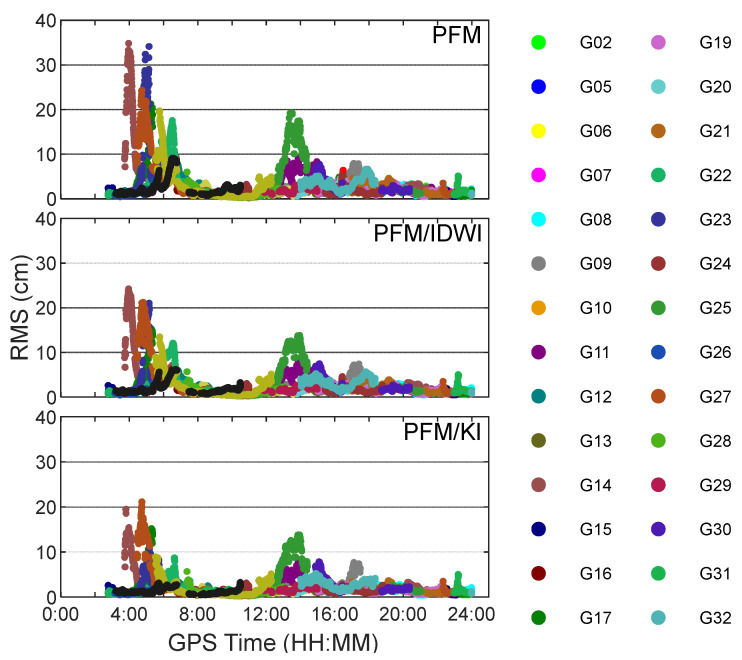
Root mean squares (RMSs) of ionospheric modeling residuals for all satellites at all reference stations.

When applying the PFM/KI model, each satellite has six polynomial fitting model coefficients and *N* residual term weight coefficients. The model and weight coefficients can be used to calculate the epoch-wise slant ionospheric delay at the user’s position for each satellite. Since the observation residuals can reflect the internal coincidence between the modeled and extracted slant ionospheric delays at reference stations, Figure 4 has been produced to show the residual distributions for the PFM, PFM/IDWI, and PFM/KI models. It can be seen that the residual distribution of the PFM/KI models is more concentrated near the zero axis. The three models’ residuals of smaller than 5 cm account for 92%, 94%, and 97%, respectively. For each satellite at all reference stations, the internal coincidence precision is epoch-wisely represented by the root mean square (RMS). The RMSs of residuals for the three models are shown in Figure 5, where different colors represent different satellites. Generally, the RMSs are mostly smaller than 10 cm, suggesting that most of the ionospheric delays have been well-modeled. The epoch-averaged RMSs are 2.7 cm, 2.3 cm, and 1.9 cm for the PFM, PFM/IDWI, and PFM/KI models, respectively. The peaks occur in the GPS time period of 4:00–6:00 (local time 12:00~14:00) because the ionosphere is more active at the local time of noon and induces larger modeling errors. In the extraction of the slant ionospheric delay at reference stations, the UPD product that is used in PPP-AR is generated after the GPS time of 2:00 to ensure that the UPD parameter is reliable. In addition, PPP-AR takes a period to converge before the precise slant ionospheric delay can be extracted at the reference stations. Thus, the RMS statistical calculation starts at the GPS time of 2:45.

**Figure 6:**
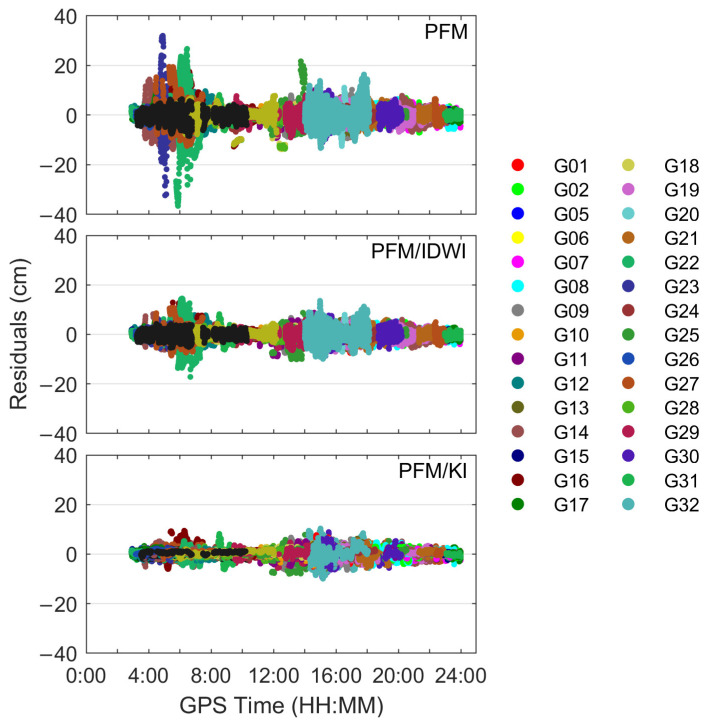
Ionospheric delay modeling errors for all satellites at all user stations.

**Figure 7:**
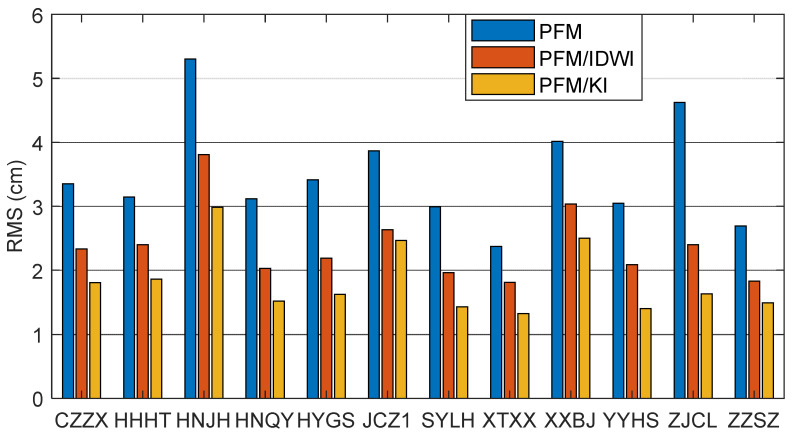
RMS of modeled ionospheric delay errors for all satellites at each user station.

To reflect the external coincidence, Figure 6 shows the time series of ionospheric modeling errors, comparing those with the extracted ionospheric delays for all satellites at all user test stations. Different colors represent different satellites. Similar to Figure 5, the modeling errors are larger for the PFM in the GPS time period of 4:00~6:00 (local time 12:00~14:00), which is caused by the active ionospheric activity. The PFM/IDWI model mitigates the ionospheric delay error more than the PFM model, and the PFM/KI model has a further improvement, with most modeling errors smaller than 10 cm. Figure 7 further shows the RMS of the modeled ionospheric delay errors for all satellites at each user station. The RMS errors at all user stations are less than 6 cm. The average RMS error for the PFM/KI model is 1.8 cm, which is improved by about 48% and 23% over the PFM-only and PFM/IDWI models, respectively. In summary, the PFM/KI modeling approach achieves the optimal performance.

### 4.3. PPP-RTK Performance Assessment

After acquiring the ionospheric delay information from the server, the user’s receiver can conduct PPP-RTK positioning. The external ionospheric delay information is used as pseudo-observables with a proper weight in an additional observation equation to constrain the ionospheric delay parameter in the UDUC PPP. The weighting scheme is provided in Table 1.

**Figure 8:**
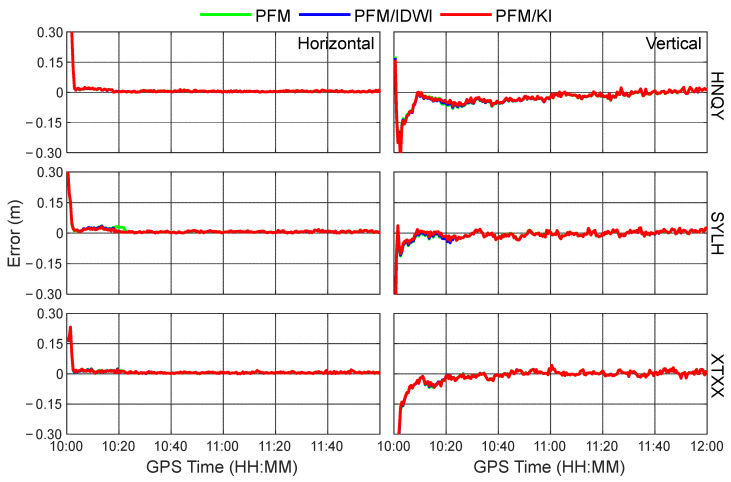
Time series of kinematic precise point positioning (PPP) errors at HNQY, SYLH, and XTXX using PFM-only, PFM/IDWI, and PFM/KI ionospheric models during the GPS time period from 10:00 to 12:00.

**Figure 9:**
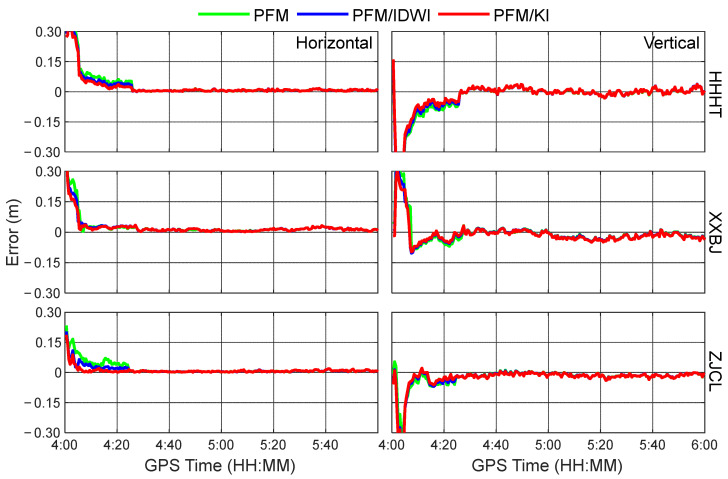
Time series of kinematic PPP errors at HHHT, XXBJ, and ZJCL using PFM-only, PFM/IDWI, and PFM/KI ionospheric models during the GPS time period from 4:00 to 6:00.

To comparatively analyze the positioning performance levels with different regional ionospheric models, simulated kinematic PPP tests using the PFM-only, PFM/IDWI, and PFM/KI regional ionospheric models at 12 user stations were carried out. As a representative, the time series of positioning errors in the horizontal and vertical directions at the test stations of HNQY, SYLH, and XTXX in the GPS time period of 10:00~12:00 are shown in Figure 8. It can be seen that the three ionospheric models do not differ much in the convergence speed of positioning solutions in the kinematic PPP. This is because the three models have smaller accuracy differences during this period, as can be seen in Figure 5 and Figure 6. Given that the ionosphere is more active at noon, Figure 9 further shows the time series of positioning errors at the three stations of HHHT, XXBJ, and ZJCL, located at the edge of the area, from GPS time 4:00 to 6:00 (local time 12:00 to 14:00). Due to a significant modeling accuracy difference during this period, the PPP convergence processes are different when using the three different ionospheric modeling approaches. Specifically, the PFM/KI and PFM/IDWI models can compensate for the ionospheric delay residuals well, and thus their positioning performances are better than the PFM-only model in the first half an hour. Furthermore, the PFM/KI outperforms the PFM/IDW slightly in the convergence process of PPP solutions, which is especially obvious in the horizontal direction at the stations ZJCL and HHHT. It is worth noting that only GPS datasets are available from Hunan CORSs, and the number of GPS satellites is usually 6~11, with an average of 8. More satellite observations will contribute to speeding up the convergence of the PPP-RTK solutions.

**Figure 10:**
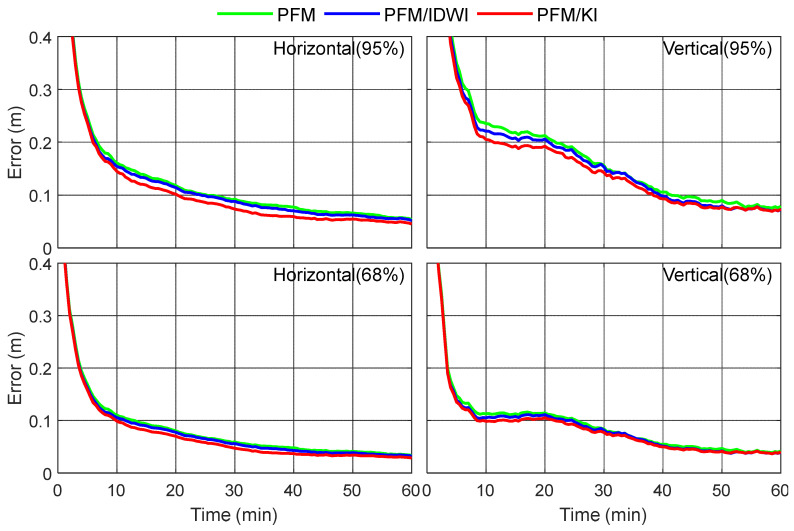
Statistical results of kinematic PPP errors using PFM-only, PFM/IDWI, and PFM/KI ionospheric models. The top and bottom panels are the 95th and 68th percentile results, respectively.

To statistically analyze the PPP performance levels when using the three different ionospheric models, the daily datasets starting from 3:00 were divided into 21 sessions for PPP processing with a session length of 1 h. The positioning errors of all sessions at all 12 test stations were used to obtain a statistical result. Figure 10 shows the positioning errors when using the three different regional ionospheric models at the confidence levels of 68% and 95%, respectively. As can be seen from Figure 10, the positioning errors when using the PFM/KI model are obviously smaller than those for the PFM-only and PFM/IDWI models after five minutes. The RMS statistical results for positioning errors and convergence times are further provided in Table 2. The convergence is defined as the time to reach a positioning error of less than 20 cm and remain within 20 cm for the subsequent epochs in kinematic PPP. Using the PFM/KI model, the convergence time reaches 1.8 min and 4.0 min in the horizontal and vertical directions with a converged positioning accuracy of 1.3 cm or 2.5 cm. Compared with the PFM-only and PFM/IDWI models, the convergence time is improved by 18% or 14% in the horizontal direction, and 9% or 5% in the vertical direction, respectively.

### 4.4. Discussion

In Section 4, we have evaluated the accuracy of the two-step PFM/KI ionospheric model and its improvement of positioning performance with comparisons to the PFM-only and PFM/IDWI models. According to the evaluation results for ionospheric models, the two-step PFM/IDWI and PFM/KI models achieve significantly higher accuracy than the one-step PFM model, which suggests that the additional interpolation can compensate for the residual errors well. Furthermore, the PFM/KI model differs from the PFM/IDWI model in terms of the interpolation method. Since the KI method takes into account the spatial correlation and variability of the ionospheric TEC, a slight accuracy improvement can be made over the IDWI method. According to the evaluation results of positioning performance, it is obvious that the positioning performance can be improved after the ionospheric TEC is better modeled. However, if the improvement of the ionospheric modeling accuracy is not considerable when using different ionospheric models, the corresponding improvement in the PPP-RTK performance is unremarkable. In this study, the Hunan CORSs with inter-station distances of 20~70 km were used for experimental tests. When the inter-station distance was increased, the ionospheric correlation between stations decreased. In this case, the two-step ionospheric modeling approach was expected to have the greater benefit.

## 5. Conclusions

Precise ionospheric delay correction is vital in PPP-RTKs. In this study, considering the complexity of spatial ionospheric variation, a two-step regional ionospheric modeling approach has been proposed by combining the PFM and KI. First, the PFM is used to model the trend term of the ionospheric delay variations. Then, the KI method is used to compensate for the unmodeled part in the PFM. Datasets at 103 reference stations in Hunan Province of China on 31 December 2018 were used to evaluate the two-step PFM/KI ionospheric modeling accuracy, with a comparison to the PFM and PFM/IDWI models. The experimental results showed that the inner residuals of less than 5 cm accounted for 92%, 94%, and 97% of the PFM, PFM/IDWI, and PFM/KI models, respectively. The evaluation results of the PFM/KI model at 12 user stations indicated that the average RMS of the ionospheric delay errors was 1.8 cm, which was reduced by about 48% and 23% when compared to the PFM and PFM/IDWI models, respectively.

In terms of the PPP-RTK performance, the kinematic PPP with the PFM/KI model converged in 1.8 min or 4.0 min with a positioning accuracy of 1.3 cm or 2.5 cm in the horizontal and vertical directions, respectively. The convergence times were improved by 18% and 14% in the horizontal direction, and 9% and 5% in the vertical direction, when compared with the positioning solutions using the PFM-only and PFM/IDWI models, respectively. It should be noted that all results analyses were carried out based on the Hunan CORS datasets, where only GPS observations are available. More experiments will be conducted to test the two-step PFM/KI regional ionospheric modeling approach in the future.

## Figures and Tables

**Table 1:** PPP data-processing strategy at the server and user sides.

Item	Server	User
Frequency	L1, L2, L5	L1, L2
Variance of observations	$\sigma_{P}^{2}=\frac{0.3^{2}}{\sin^{2}\left(el\right)},\;\sigma_{L}^{2}=\frac{0.003^{2}}{\sin^{2}\left(el\right)\;}$ , where el is elevation	Same as server
Elevation cutoff angle	7.5°	Same as server
Satellite orbit and clock	Products from Center for Orbit Determination	Same as server
Antenna phase center offsets and variations	igs14_2035.atx	Same as server
Differential code bias	Products from Chinese Academy of Sciences	Same as server
Receiver coordinates	Estimated as constants	Estimated as white noise
Tropospheric delay	Dry component corrected by Saastamoinen model with atmospheric pressure $p=1013.25\cdot {\left(1-2.2557\times 10^{-5}H\right)}^{5.2568}$, where H is the altitude of the station; zenith wet delay estimated as a random walk	Same as server
Ionospheric delay	Estimated as a random walk	Pseudo-observable variances: $\sigma_{∆I}^{2}=0.2^{2}$
Receiver clock Ambiguities	Estimated as white noise Estimated as constants	Same as server Same as server

**Table 2:** Average convergence times and RMSs of positioning errors using three different regional ionospheric models.

Ionospheric Model	Convergence Time (min)		RMS (cm)	
	Horizontal	Vertical	Horizontal	Vertical
PFM	2.2	4.4	1.4	2.6
PFM/IDWI	2.1	4.2	1.4	2.5
PFM/KI	1.8	4.0	1.3	2.5

## Data Availability

The precise GNSS satellite orbit, clock, and DCB products are available at https://cddis.nasa.gov/archive/gnss/ (accessed on 30 December 2023).
